# Myocardial Fatty Foci in Tuberous Sclerosis Complex: Imaging Findings

**DOI:** 10.7759/cureus.693

**Published:** 2016-07-14

**Authors:** Jeremy Burt, Baiywo Rop, Edward Derrick, Jamil Armaly, Usman Siddiqui

**Affiliations:** 1 Diagnostic Radiology, Florida Hospital-Orlando; 2 Diagnostic Radiology, Florida Hospital Orlando; 3 Cardiology, Florida Hospital-Orlando

**Keywords:** tuberous sclerosis complex, myocardial fatty foci, rhabdomyoma, cardiac lipoma, cardiac imaging, cardiac mri

## Abstract

Tuberous sclerosis complex (TSC) is a rare autosomal dominant genetic syndrome. The hallmark of the disease is multiple hamartomatous lesions in multiple organ systems. Common cardiac manifestations of TSC are rhabdomyomas, which are a benign tumor of striated muscle. In some patients with TSC, myocardial fatty foci (MFF) deposition has been described with or without the presence of rhabdomyomas.

We present the case of a 24-year-old female with TSC and refractory seizures, who was evaluated with cardiac magnetic resonance (CMR) for an intracardiac right ventricular mass thought to be rhabdomyoma on echocardiography and for multiple areas of myocardial fatty deposition. Myocardial fatty deposition is a common finding in patients at cardiac imaging. In patients with TSC, it is critical that fatty deposits and lipomas are clearly distinguished from rhabdomyoma. CMR is an integral part of characterizing cardiac masses as it has superior soft tissue characterization and a wider field of view compared to echocardiography. A positive correlation has been shown between the number of MFF and the degree of extracardiac tuberous sclerosis (TS) manifestations suggesting that MFF may indicate more severe multiorgan disease in patients with TSC.

Cardiac MR is superior to echocardiogram in evaluating and distinguishing intracardiac lipomas and fatty deposits from rhabdomyomas. Published studies have indicated that in patients with TSC, the presence of MFF correlates with the severity of multiorgan disease as was seen in our case.

## Introduction

Tuberous sclerosis complex (TSC) is a rare autosomal dominant genetic syndrome of variable phenotypes arising due to mutation of cellular proliferation genes TSC1 or TSC2. This leads to deregulation of cellular proliferation protein mammalian target of rapamycin (mTOR), resulting in cellular hyperproliferation [1]. The hallmark of the disease is multiple hamartomatous lesions in a variety of organ systems. Most of the rhabdomyomas are detected during fetal life or infancy. Common cardiac manifestations of TSC are rhabdomyomas, which are a benign tumor of striated muscle. Although up to 80% of these tumors are asymptomatic, some may cause ventricular outlet obstruction or arrhythmias [2]. In some patients with TSC, myocardial fatty foci (MFF) deposition has been described with or without the presence of rhabdomyomas [3-4]. A limited number of studies found a radiology-pathology correlation indicating these MFF either represent intramyocardial angiomyolipomas or lipomas [5-6]. Two studies have suggested a linear correlation of MFF with severity of multiorgan involvement and that identification of MFF on cardiac imaging should prompt further evaluation for TSC [7-8]. Informed consent from the patient was not required for this study.

## Case presentation

A 24-year-old female presented to our hospital with refractory seizures. The patient had been diagnosed with TSC at the age of seven months, outside of the United States. Manifestations of the disease in this patient included bilateral renal angiomyolipomas treated with embolization, lymphangioleiomyomatosis (LAM), and subependymal giant cell astrocytoma (SEGA).

**Figure 1:**
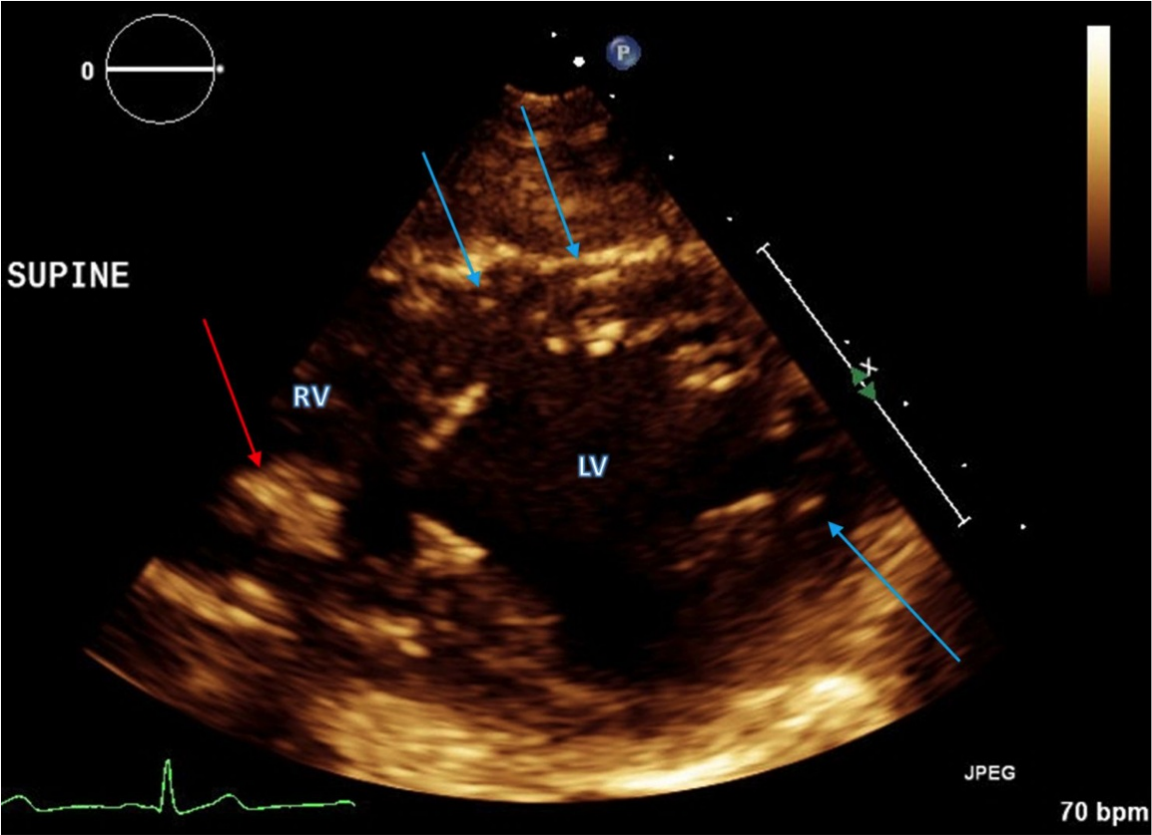
Transthoracic Echocardiogram of a Patient with Tuberous Sclerosis Complex Transthoracic echocardiogram in short axis demonstrates an echogenic mass in the midseptal region of the right ventricle (red arrow). Additional punctate echogenic foci are also identified (blue arrows). RV = right ventricle; LV = left ventricle.

Screening transthoracic echocardiography (TTE) (including 2D M-mode spectral and color Doppler echocardiography) demonstrated normal global left ventricular wall motion, contractility, and ejection fraction. An echogenic mass, initially thought to be a rhabdomyoma, was noted within the right ventricle (RV) at the mid septal region (Figure 1). There were also multiple punctate echogenic foci scattered in the myocardium. Cardiac MR (CMR) was obtained for further characterization of the RV mass and echogenic foci. An unenhanced chest computed tomography (CT) scan obtained a few months prior for evaluation of a pneumothorax related to LAM was used for comparison.

**Figure 2:**
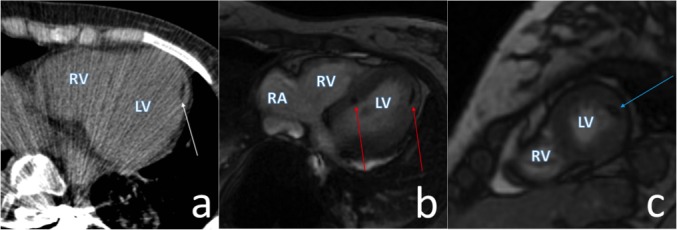
Cardiac CT and MR of Myocardial Fatty Foci (a) Axial noncontrast CT of the heart shows mid myocardial MFF deposit in the lateral wall (white arrow). (b) A bSSFP cardiac MR image in a four-chamber horizontal long axis view demonstrates myocardial focal fatty deposits with associated chemical shift artifact (red arrows). (c) Similar findings demonstrated on the two-chamber short axis view (blue arrow). bSSFP = balanced stready-state free precession; RA = right atrium; RV = right ventricle; LV = left ventricle.

**Figure 3:**
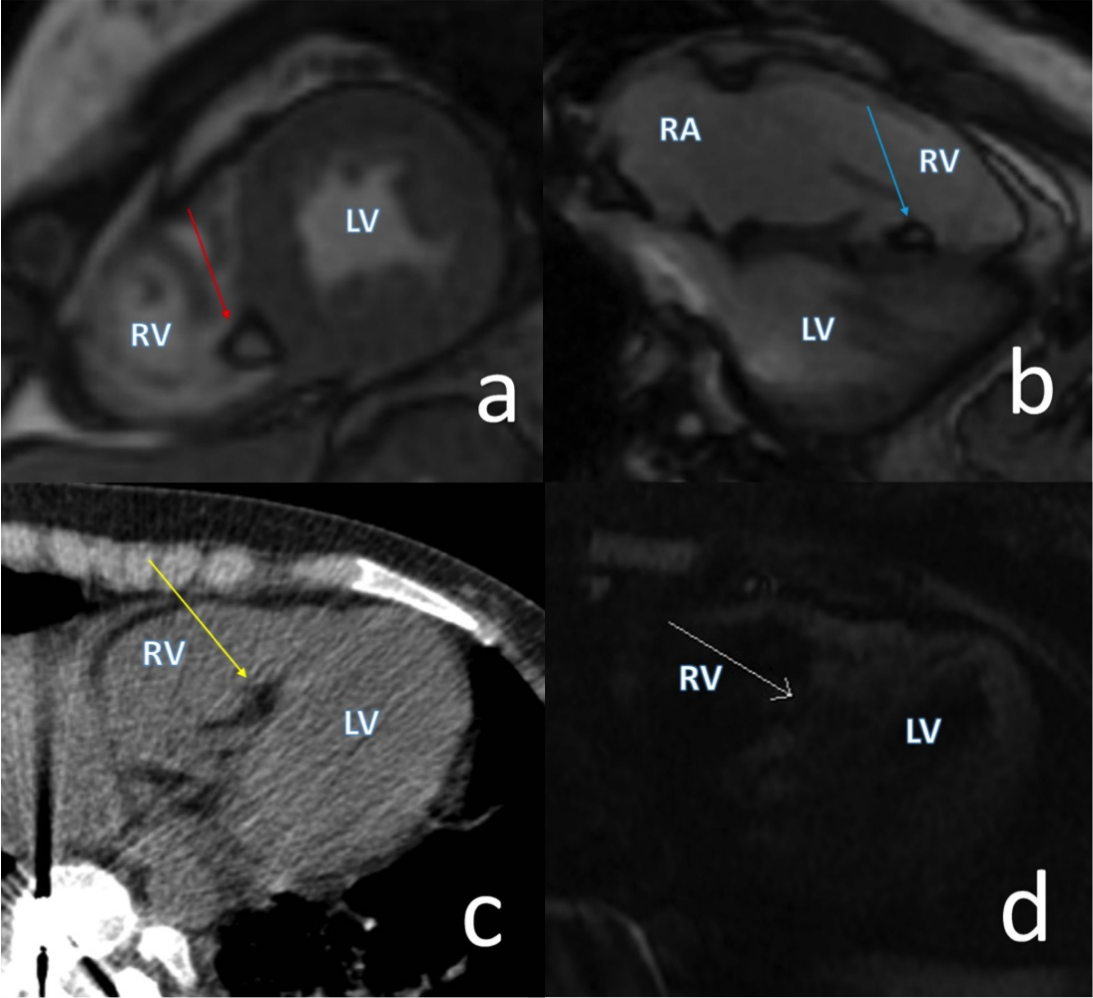
Cardiac CT and MR of an Intracardiac Lipoma (a) A bSSFP cardiac MR image in a two-chamber short axis view demonstrates a subendocardial focal fatty deposit in the midportion of the interventricular septum with associated chemical shift artifact (red arrow). (b) Similar finding demonstrated on the bSSFP four-chamber horizontal long axis view (blue arrow) and (c) Unenhanced axial CT (yellow arrow). (d) Axial TIR (fat saturated) image demonstrates loss of signal in the subendocardial deposit (white arrow). bSSFP = balanced stready-state free precession; TIR = triple inversion recovery; RA = right atrium; RV = right ventricle; LV = left ventricle.

A multiplanar contrast-enhanced CMR demonstrated normal cardiac anatomy. At least 10 definitive areas of MFF were identified within the midwall of the interventricular septum, lateral wall, moderator band, and septal leaflet attachment site. The largest midwall deposit was in the lateral wall measuring 8 x 5 mm (Figure 2). The MFF lesions identified on CMR correlated geographically to the echogenic foci seen on the prior TTE. There was also a large fatty deposit in the subendocardial region of the right ventricle along the mid interventricular septum compatible with a lipoma (Figure 3). This lesion and the other MFF lesions had no enhancement and no internal soft tissue components. The cardiac indices, cardiac valves, and great vessels were all normal. There was no evidence of cardiac rhabdomyoma or thrombus.

## Discussion

Myocardial fatty deposition is a common finding at cardiac imaging, especially in patients of advanced age. Pathologic conditions with myocardial fat deposition include physiologic fat, healed myocardial infarction (MI), arrhythmogenic right ventricular cardiomyopathy (ARVC), dilated cardiomyopathy, and muscular dystrophy. Distinction of these different entities can be made using the patient’s age, clinical history, ventricular size and function, myocardial thickness, location of fat deposition, and regional wall motion abnormalities [9]. In patients with TSC, it is critical that fatty deposits and lipomas are clearly distinguished from rhabdomyoma. Cardiac rhabdomyomas are the commonest cardiac tumors in patients with TSC, usually presenting as multiple, or occasionally single, large hyperechoic ventricular mass lesions of varying sizes on echocardiography [8]. Unfortunately, fatty deposits and lipomas associated with TSC also manifest as well-circumscribed hyperechoic lesions on echocardiography [5, 10].

CMR imaging is an integral part of characterizing cardiac masses. CMR has superior soft tissue characterization and a wider field of view compared to echocardiography. Additional anatomic pre-surgical information such as mass mobility, valvular involvement, and possible myocardial and extracardiac extension can be obtained using CMR. Contrast-enhanced CMR imaging is also useful in defining the margins of the tumor, characterizing the mass, and distinguishing from cardiac thrombus.

Rhabdomyomas on CMR are usually isointense to myocardium on T1-weighted images although they can be slightly hyperintense. They are hyperintense on T2-weighted images and demonstrate hypoenhancement relative to myocardium on contrast-enhanced images [10]. In contrast, MFF are well-circumscribed foci of fat easily distinguished on balanced steady-state free precession or bSSFP-weighted imaging by a rim of chemical shift artifact, which is absent in rhabdomyomas. MFF have high T1- and T2-signal, have no contrast enhancement, and lose signal on triple inversion recovery (TIR) or other fat saturated sequences. Similarly, cardiac lipomas appear as nonenhancing, T1-hyperintense and T2-hyperintense homogenous encapsulated masses protruding into the cardiac chamber or pericardial space [9].

**Figure 4:**
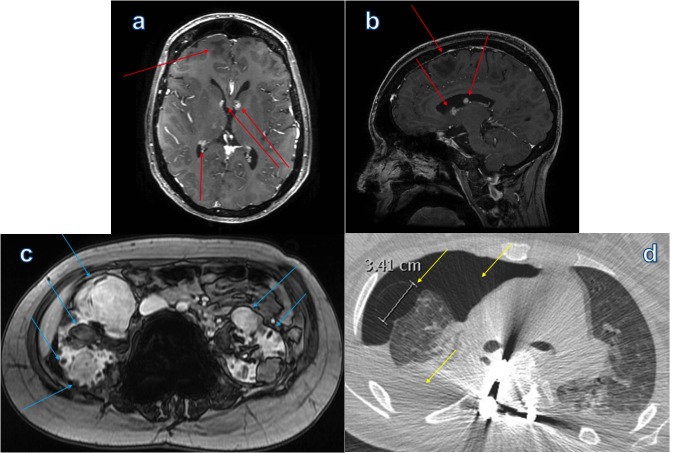
Montage of CT and MR Findings in Tuberous Sclerosis Complex CT and MR images demonstrate manifestations of multiorgan disease related to tuberous sclerosis complex. (a, b) Contrast-enhanced, T1-weighted brain MRI in axial and sagittal planes show subependymal tubers and signal abnormality in the subcortical white matter (red arrows). (c) Axial out-of-phase T1-weighted images show innumberable angiomyolipomas scattered throughout both kidneys (blue arrows). (d) Axial chest CT demonstrates a prominent lung cyst in the anterior right middle lobe from lymphangioleiomyomatosis, a large pneumothorax, and right-sided chylous effusion (yellow arrows).

A positive correlation has been shown between the number of MFF and the degree of extracardiac TS manifestations. This suggests that the presence of MFF may indicate more severe multiorgan disease in patients with TSC [7]. Our patient had severe cranial, pulmonary, renal, and cardiac manifestations of TSC (Figure 4).

## Conclusions

We report a case of myocardial fatty foci and subendocardial lipoma diagnosed using cardiac magnetic resonance imaging in a patient with tuberous sclerosis complex. Similar to previous reports of myocardial fatty foci, our patient had multiple intracardiac fatty lesions in addition to significant multiorgan disease related to tuberous sclerosis complex. We demonstrate that, although difficult to definitively characterize on echocardiography, myocardial fatty foci and intracardiac lipoma can be easily distinguished from rhabdomyoma using cardiac magnetic resonance imaging.
